# Germacrone reverses Adriamycin resistance through cell apoptosis in multidrug-resistant breast cancer cells

**DOI:** 10.3892/etm.2014.1932

**Published:** 2014-08-26

**Authors:** XIAO-HONG XIE, HONG ZHAO, YUAN-YUAN HU, XI-DONG GU

**Affiliations:** Department of Breast Surgery, Zhejiang Provincial Hospital of Traditional Chinese Medicine, Hangzhou, Zhejiang 310014, P.R. China

**Keywords:** germacrone, human breast cancer, multidrug resistance

## Abstract

Multidrug resistance (MDR) is a major obstacle to the chemotherapeutic treatment of breast cancer. Germacrone, the main component of Rhizoma Curcuma, has been shown to possess antitumor, anti-inflammatory and immunomodulatory properties. The aim of the present study was to investigate the effect of germacrone on MCF-7/Adriamycin (ADR) multidrug-resistant human breast cancer cells. The treatment of MCF-7/ADR cells with a combination of germacrone and ADR resulted in an increase in cytotoxicity compared with that of ADR alone, as determined using an MTT assay. Flow cytometric analysis revealed that germacrone promoted cell apoptosis in a dose-dependent manner, whilst treatment with germacrone plus ADR enhanced the apoptotic effect synergistically. Furthermore, the results from the western blot analysis demonstrated that augmenting ADR treatment with germacrone resulted in a reduction of anti-apoptotic protein expression levels (bcl-2) and enhancement of pro-apoptotic protein expression levels (p53 and bax) in MCF-7/ADR cells compared with the levels achieved by treatment with ADR alone. In addition, germacrone significantly reduced the expression of P-glycoprotein via the inhibition of the multidrug resistance 1 (MDR1) gene promoter. These findings demonstrate that germacrone has a critical role against MDR and may be a novel MDR reversal agent for breast cancer chemotherapy.

## Introduction

Breast cancer has emerged as the most common malignancy observed in females worldwide. It is the leading cause of cancer mortalities among females, accounting for 23% of total cancer cases and 14% of cancer mortalities (1). Chemotherapy is one of the main treatments for patients diagnosed with breast cancer. However, resistance to chemotherapeutic drugs has gradually emerged (2). Resistance to chemotherapeutics, in particular, multidrug resistance (MDR), remains the leading cause of chemotherapy failure. The MDR mechanism is an extremely complicated process, involving drug metabolic biotransformation, drug efflux increase and alteration of the repair ability for anticancer drug-induced DNA damage (3,4). Therefore, it is imperative to find novel and effective strategies to reverse drug resistance.

At present, the main strategy for overcoming MDR is to use sensitizing or reversal agents, combined with chemotherapeutic drugs (5). Traditional Chinese herbs are a significant source of drugs that serve as potential therapeutic compounds for cancer treatment (6). Numerous studies have identified reversal agents from natural products. Rhizoma Curcuma is a widely used traditional herb for antitumor therapy in China and other Asian countries (7). Germacrone, the main component of Rhizoma Curcuma, has been shown to possess antitumor, anti-inflammatory and immunomodulatory properties. A recent study demonstrated that treatment of the hepatoma cell lines HepG2 and Bel7402 with germacrone promoted cell apoptosis, associated with the upregulation of bax and the downregulation of bcl-2, indicating that germacrone may have a potential role in the treatment of hepatocellular carcinoma (8). Germacrone has also been found to inhibit the proliferation of the breast cancer cell lines MCF-7 and MDA-MB-231 by inducing G0/G1 and G2/M cell cycle arrest and apoptosis through the mitochondria-mediated caspase pathway (9). However, the function of germacrone on MDR in human breast cancer has not yet been investigated. Therefore, the present study aimed to investigate the effect of germacrone on MCF-7/Adriamycin (ADR) multidrug-resistant human breast cancer cells.

## Materials and methods

### Reagents

Germacrone and ADR were purchased from Sigma (St. Louis, MO, USA). RPMI-1640 culture medium, fetal bovine serum (FBS), phosphate-buffered saline (PBS), penicillin-streptomycin and 0.25% (w/v) trypsin/1 mM EDTA were purchased from Gibco (Grand Island, NY, USA).

### Cell culture

MCF-7 and MCF-7/ADR human breast cancer cells were purchased from the Chinese Academy of Sciences (Shanghai, China) and were maintained in RPMI-1640 medium containing 10% (v/v) FBS, 100 U/ml penicillin and 100 μg/ml streptomycin at 37°C in a humidified 5% CO_2_ incubator. MCF-7/ADR cells were cultured in the medium containing 1 μg/ml ADR in order to maintain the MDR phenotype, and were then maintained in drug-free medium for at least two days prior to use.

### Cell proliferation assay

Cell proliferation was analyzed using the MTT assay. In brief, MCF-7 and MCF-7/ADR cells were independently seeded at a density of 3×10^4^ cells/well into 96-well plates and left to adhere overnight. The cells were then incubated with 0–125 μmol/l ADR, 0–250 μmol/l germacrone or a combination of 0–250 μmol/l germacrone and 1 μmol/l ADR for 48 h. A total of 10 ml 5 mg/ml MTT was added and the cells were incubated in the dark at 37°C for 2 h. The absorbance was then determined at a wavelength of 492 nm (Bio-Rad Laboratories, Inc., Hercules, CA, USA).

### Apoptosis assay

MCF-7/ADR cells were seeded in 12-well plates and treated with different concentrations (0, 50, 150 and 250 μmol/l) of germacrone and/or 1.0 μmol/l ADR for 48 h. The apoptotic morphology of the cells was evaluated using hematoxylin and eosin staining for visualization under a light microscope (Leica Microsystems, Wetzlar, Germany; magnification, ×200). Cells undergoing apoptosis were assessed using an Annexin V-FITC/PI Apoptosis Detection kit, in accordance with the manufacturer’s instructions (BD Biosciences, Franklin Lakes, NJ, USA). The number of apoptotic cells was quantified using a flow cytometer (FACSCalibur™; BD Biosciences) and analyzed using CellQuest software (BD Biosciences).

### Western blot analysis

For the western blot analysis of total cell lysates, cells were harvested and washed with ice-cold PBS. The protein concentration in the lysates was measured using a BCA Protein assay kit (Thermo Fisher Scientific, Rockford, IL, USA) in accordance with the manufacturer’s instructions. Cell lysate samples (50 μg per lane) were separated using 10% SDS-PAGE and transferred to polyvinylidene difluoride membranes (Millipore, Billerica, MA, USA). Membranes were incubated overnight at 4°C with antibodies against p53, bax, bcl-2, P-gp and GAPDH (Cell Signaling Technology, Inc., Danvers, MA, USA). Membranes were washed three times with Tris-buffered saline with Tween^®^ 20 and incubated for 1 h at room temperature with the appropriate secondary antibody (Cell Signaling Technology, Inc.). Immunoreactive bands were detected using the Enhanced Chemiluminescence kit for Western blotting detection and using a ChemiGenius bioimaging system (Syngene, Frederick, MD, USA).

### Quantitative PCR (qPCR)

The mRNA expression of MDR1 was analyzed by qPCR. Total RNA was extracted from the treated MCF-7/ADR cells using the RNeasy kit with the DNase set (Qiagen GmbH, Hilden, Germany). For cDNA synthesis, the template was reverse transcribed using SuperScript II RNase H-reverse transcriptase and oligo(dT)_25_ as a primer (Invitrogen Life Technologies, Carlsbad, CA, USA). PCR was carried out under the following conditions: an initial stage of 95°C for 30 sec, then a two step program of 95°C for 5 sec and 60°C for 31 sec over 40 cycles and was performed in triplicate. The relative target mRNA levels were analyzed with ABI Prism 7300 software (Applied Biosystems, CA, USA) and normalized against that of the internal control, GAPDH.

### Dual luciferase assay

MCF-7/ADR cells were seeded in 96-well plates for 24 h until they reached 90–95% confluence at the time of transfection. The cells were co-transfected with the MDR1 promoter recombinant vector pGL3-basic-MDR1, and a control vector according to the manufacturer’s instructions (Promega Corporation, Madison, WI, USA). The cells were collected 48 h after transfection and analyzed using a Dual-Luciferase Reporter assay system (Promega Corporation).

### Statistical analysis

For statistical analysis, all data were analyzed using SPSS statistical software, version 13.0 (SPPS, Inc., Chicago, IL, USA), and are presented as the mean ± standard deviation. Comparisons between groups were performed using an analysis of variance. P<0.05 was considered to indicate a statistically significant difference.

## Results

### Effect of germacrone on ADR resistance in breast cancer cells

**Figure 1:**
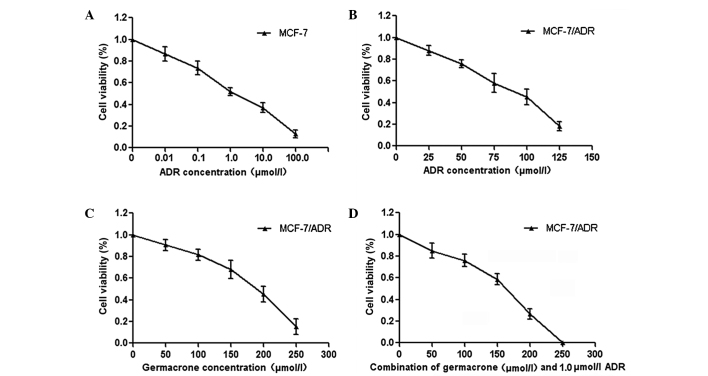
Effect of germacrone on ADR resistance in breast cancer cells. The MTT assay was used to measure the cytotoxicity of (A) ADR in MCF-7 cells and (B) ADR, (C) germacrone and (D) combination of ADR and different concentrations of germacrone in MCF-7/ADR cells. Data are presented as the mean ± standard deviation. Each experiment was repeated at least three times. ADR, Adriamycin.

The cytotoxicity of germacrone and ADR in the MCF-7/ADR human breast cancer cell line was analyzed using the MTT assay. The results showed that the IC_50_ of ADR at 48 h was 1.27±0.12 μmol/l in MCF-7 cells and 87.40±5.24 μmol/l in MCF-7/ADR cells (Fig. 1A and B). In addition, the IC_50_ of germacrone was 180.41±12.45 μmol/l in MCF-7/ADR cells following 48 h of treatment. The MTT assay demonstrated that germacrone treatment inhibited cell viability in a concentration-dependent manner (Fig. 1C). Furthermore, treatment with a combination of germacrone and ADR inhibited cell viability synergistically (Fig. 1D). In combination, these results demonstrate that the treatment of MCF-7/ADR cells with a combination of germacrone and ADR results in an increase in cytotoxicity.

### Germacrone promotes the rate of apoptosis induced by ADR in MCF-7/ADR cells

**Figure 2:**
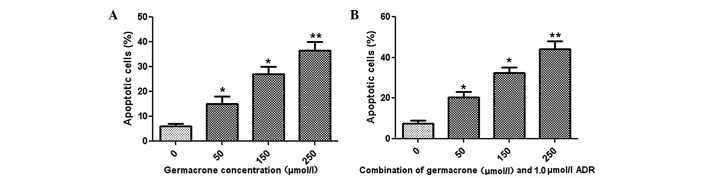
Effect of germacrone on the apoptosis rate induced by ADR in MCF-7/ADR cells. Flow cytometry was used to analyze the cell apoptosis rate following the administration of different concentrations of germacrone (A) alone or (B) in combination with 1.0 μmol/l ADR. ^*^P<0.05, ^**^P<0.01 compared with the control group treated with ADR (1.0 μmol/l) alone. Data are presented as the mean ± standard deviation. Each experiment was repeated at least three times. ADR, Adriamycin.

Flow cytometry was used to measure the effects of germacrone and ADR on the apoptosis rate in MCF-7/ADR cells. The results revealed that germacrone treatment promoted cell apoptosis in a concentration-dependent manner in MCF-7/ADR cells (Fig. 2A). Furthermore, treatment with a combination of ADR (1.0 μmol/l) and germacrone at different concentrations (50, 150 or 250 μmol/l) caused a significant increase in the apoptotic rate in the MCF-7/ADR cells (Fig. 2B).

### Effect of germacrone and ADR on apoptotic proteins in MCF-7/ADR cells

**Figure 3:**
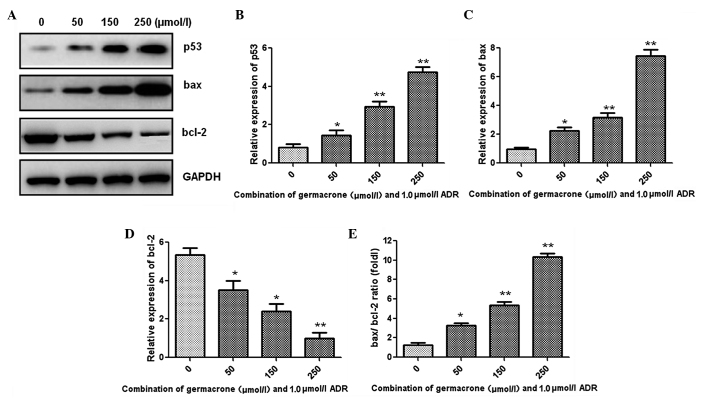
Effect of germacrone and ADR on apoptosis-associated proteins in MCF-7/ADR cells. MCF-7/ADR cells were treated with ADR (1.0 μmol/l) and germacrone at different concentrations and subjected to western blot analysis. (A) Expression levels of apoptosis-associated proteins were measured using antibodies against p53, bax and bcl-2. GAPDH was used as an internal control. Relative band intensities were used for quantification of (B) p53, (C) bax, (D) bcl-2 and the (E) bax/bcl-2 ratio. ^*^P<0.05; ^**^P<0.01 compared with the control group treated with ADR (1.0 μmol/l) alone. Data are presented as the mean ± standard deviation. Each experiment was repeated at least three times. ADR, Adriamycin.

Western blot analysis was performed to detect the changes in the levels of apoptosis-associated proteins, and the results are shown in Fig. 3A. Following treatment with a combination of germacrone (50, 150 or 250 μmol/l) and ADR (1.0 μmol/l), the expression of p53 and bax was significantly increased (Fig. 3B and C). Furthermore, the expression level of the anti-apoptotic protein bcl-2 was markedly decreased by treatment with the combination compared with that in the control group treated with ADR (1.0 μmol/l) alone (Fig. 3D). In addition, the bax/bcl-2 ratio was significantly increased in a concentration-dependent manner following treatment with germacrone and ADR together (Fig. 3E).

### Germacrone treatment decreased the expression of MDR1

**Figure 4:**
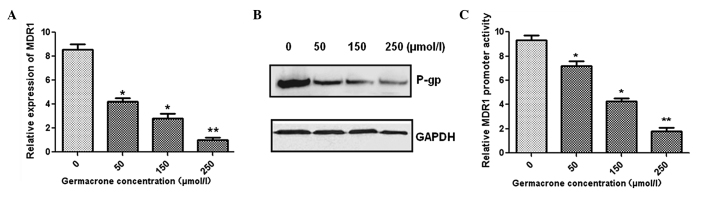
Germacrone treatment decreased the expression of MDR1. (A) Quantitative polymerase chain reaction and (B) western blot analysis were used to determine the gene expression of MDR1 and protein expression of P-gp in MCF-7/ADR cells. GAPDH was used as a protein loading control. (C) The dual luciferase assay was used to measure the MDR1 promoter activity. Data are presented as the mean ± standard deviation. Each experiment was repeated at least three times. MDR1; multidrug resistance 1; P-gp, P-glycoprotein.

In order to investigate the reversal mechanism of germacrone, MCF-7/ADR cells were treated with various concentrations of germacrone. qPCR demonstrated that MDR1 gene expression was significantly inhibited by germacrone administration in a concentration-dependent manner (Fig. 4A). In addition, the expression of P-gp was also downregulated, as shown by western blot analysis (Fig. 4B). Furthermore, the dual luciferase assay was used to measure the MDR1 promoter activity. The results demonstrated that MDR1 promoter expression levels were markedly decreased following treatment with germacrone at different concentrations compared with the levels in the control group (Fig. 4C). In combination, these results indicate that germacrone may decrease the P-gp expression levels via the inhibition of the activity of the MDR1 gene promoter.

## Discussion

Germacrone is a sesquiterpene and has been previously demonstrated to be a promising therapeutic agent against several types of cancer. A previous study showed that germacrone is able to inhibit breast cancer cell proliferation (9). However, the function of germacrone on MDR in human breast cancer has yet to be elucidated. Therefore, in the present study, the effect of germacrone on MCF-7/ADR human breast cancer multidrug-resistant cells was investigated.

The MTT assay results revealed that germacrone significantly inhibited the proliferation of MCF-7/ADR cells in a concentration-dependent manner. Treatment with a combination of germacrone and ADR effectively decreased the viability of the MCF-7/ADR cells *in vitro*. These results indicate that germacrone reversed the ADR resistance of MCF-7/ADR cells.

ADR is a widely used chemotherapy drug that induces tumor cell apoptosis; however, resistance against ADR has occurred in numerous types of tumor cells (10,11). Apoptosis is a complicated and precise process of programmed cell death, characterized by cell shrinkage, phosphatidylserine externalization and chromatin condensation (12). In the present study, flow cytometric analysis indicated that germacrone dose-dependently promoted MCF-7/ADR cell apoptosis whereas ADR did not significantly induce apoptosis in the MCF-7/ADR cells. However, treatment with ADR and germacrone markedly enhanced the apoptosis rate in the MCF-7/ADR cells.

Furthermore, the expression of apoptosis-associated proteins was determined using western blot analysis. The protein p53, encoded by the TP53 gene, has an important role in multi-cellular organisms, where it regulates cell apoptosis and cell proliferation (13,14). Bax is a p53 primary-response gene and is involved in the apoptotic induction regulated by p53. p53 directly activates the proapoptotic protein bax to permeabilize mitochondria and engage the apoptotic pathway (15,16). The anti-apoptotic protein bcl-2, which prevents disruption of the mitochondrial physiology, is a response gene of p53 and involved in p53-regulated apoptosis (17). The present study showed that treatment with germacrone and ADR significantly elevated the expression levels of p53 and bax and decreased the expression levels of the anti-apoptotic protein bcl-2.

Drug resistance in breast cancer cells is associated with increased expression of resistance proteins (18). These proteins decrease the accumulation of ADR in the cells, thereby reducing the effects of ADR (19). P-gp, encoded by the MDR1 gene, is one of the multi-drug resistance-associated proteins acting as an efflux pump (20,21). The overexpression of P-gp may lower intracellular drug accumulation and decrease the cellular toxicity of chemo-therapeutics, including ADR, epirubicin, mitoxantrone and paclitaxel (22,23). The present study demonstrated that germacrone reduced the gene and protein expression levels of P-gp in MCF-7/ADR cells, as shown by the results from the qPCR and western blot analysis. Furthermore, the activity of the MDR1 gene promoter, which mediates P-gp expression, was significantly downregulated following germacrone treatment, thereby promoting ADR-induced MCF-7/ADR cell apoptosis.

In conclusion, the present study demonstrated for the first time, to the best of our knowledge, that germacrone reverses ADR resistance through cell apoptosis in MDR breast cancer cells. Therefore, germacrone is of important clinical significance for MDR during tumor therapy and may be a novel MDR reversal agent for breast cancer chemotherapy.
